# Activation of L-lactate oxidase by the formation of enzyme assemblies through liquid–liquid phase separation

**DOI:** 10.1038/s41598-023-28040-1

**Published:** 2023-01-25

**Authors:** Tomoto Ura, Ako Kagawa, Nanako Sakakibara, Hiromasa Yagi, Naoya Tochio, Takanori Kigawa, Kentaro Shiraki, Tsutomu Mikawa

**Affiliations:** 1 Institute of Pure and Applied Sciences, University of Tsukuba, 1-1-1 Tennodai, Tsukuba, Ibaraki 305-8573 Japan; 2 RIKEN Center for Biosystems Dynamics Research, 1-7-22 Suehiro-Cho, Tsurumi-Ku, Yokohama, 230-0045 Japan

**Keywords:** Enzymes, Biochemistry, Molecular biology

## Abstract

The assembly state of enzymes is gaining interest as a mechanism for regulating the function of enzymes in living cells. One of the current topics in enzymology is the relationship between enzyme activity and the assembly state due to liquid–liquid phase separation. In this study, we demonstrated enzyme activation via the formation of enzyme assemblies using L-lactate oxidase (LOX). LOX formed hundreds of nanometer-scale assemblies with poly-L-lysine (PLL). In the presence of ammonium sulfate, the LOX-PLL clusters formed micrometer-scale liquid droplets. The enzyme activities of LOX in clusters and droplets were one order of magnitude higher than those in the dispersed state, owing to a decrease in *K*_M_ and an increase in *k*_cat_. Moreover, the clusters exhibited a higher activation effect than the droplets. In addition, the conformation of LOX changed in the clusters, resulting in increased enzyme activation. Understanding enzyme activation and assembly states provides important information regarding enzyme function in living cells, in addition to biotechnology applications.

## Introduction

Enzymes are observed in various assembly states in living systems. For example, enzymes exist in aggregate^1,2^, fibril^3,4^, and condensate^5,6^ states in response to stress. Hence, it is assumed that the function of enzymes is precisely controlled by their assembly states. However, there is no unified view of the driving force of these assemblies and their effect on enzyme activity^7^. This may be because historically, several in vitro enzymatic studies have been performed under conditions that do not promote enzyme assembly, such as using diluted and purified enzymes.

In the last decade, liquid–liquid phase separation (LLPS) is one of the subjects of debate in cell biology^8,9^. LLPS is a phenomenon in which a one-phase solution changes to a two-phase solution according to thermodynamic equilibrium^8,9^. Liquid droplets formed by LLPS can regulate enzyme activities^10–12^. Extensive studies on intercellular droplets reveals the importance of intrinsically disordered proteins (IDPs) or nucleic acids in droplet formation^13–15^. IDPs comprise intrinsically disordered regions (IDRs) that lack a fixed three-dimensional structure^16^. Some enzymes interact favorably with IDPs and nucleic acids, known as droplet scaffold molecules. For example, RubisCO, a well-known enzyme of more than 500 kDa for carbon dioxide fixation, functions by forming droplets with a small IDP^17^. Multienzyme assemblies that activate multistep reactions also exhibit liquid-like properties and require IDP domains^18^. Furthermore, several metabolic enzymes have RNA-binding ability^19,20^, which are prone to forming droplets. Numerous in vitro studies have revealed that enzymes such as ribozyme^21^, kinase^12,22^, multienzyme complexes^23^, RNA polymerase, and ribosomes^24^ are activated in the formation of droplets. The droplets in these studies range in size from a few to tens of µm, making them easy to detect using an optical microscope. Moreover, several in vivo and in vitro studies report the presence of submicron-sized clusters as a precursor to micron-sized droplets^25–27^. Thus, the relationship between the assembly state and biological functions, such as enzymatic activation, is therefore of interest, however, this has not been demonstrated experimentally.

In this study, we investigated the relationship between assembly state and enzyme activity using L-lactate oxidase (LOX) as a model. Poly-L-lysine (PLL), which mimics the intrinsically disordered region of IDP^28^, was used as a scaffold molecule that electrostatically interacts with LOX. The addition of small amounts of salt (< 100 mM) has been reported to promote droplet formation, presumably by modulating electrostatic interactions^29^. Furthermore, kosmotropic salts tend to promote droplet formation more efficiently^29^. When we investigated effective salts for LOX droplet formation, ammonium sulfate (NH_4_)_2_SO_4_, a kosmotropic salt, caused LOX droplets to form most efficiently as expected (Supplementary Fig. S1). Thus, in this study, we employed (NH_4_)_2_SO_4_ salt to stabilize droplets. In the presence of PLL and absence of (NH_4_)_2_SO_4_, LOX formed a soluble hundreds-nm-scale assembly, called “cluster_lox_.” The addition of a small amount of (NH_4_)_2_SO_4_ formed several visible droplets in the scale of micrometers. The activities of both the assembly states were one order of magnitude higher than that of the enzyme in the dispersed state. The clusters_lox_ and droplets have the common features of increasing *k*_cat_ and decreasing *K*_M_, however, the activation effect was higher for clusters_lox_ than for droplets.

## Results

### LOX assembly states in the presence of PLL and ammonium sulfate

**Figure 1:**
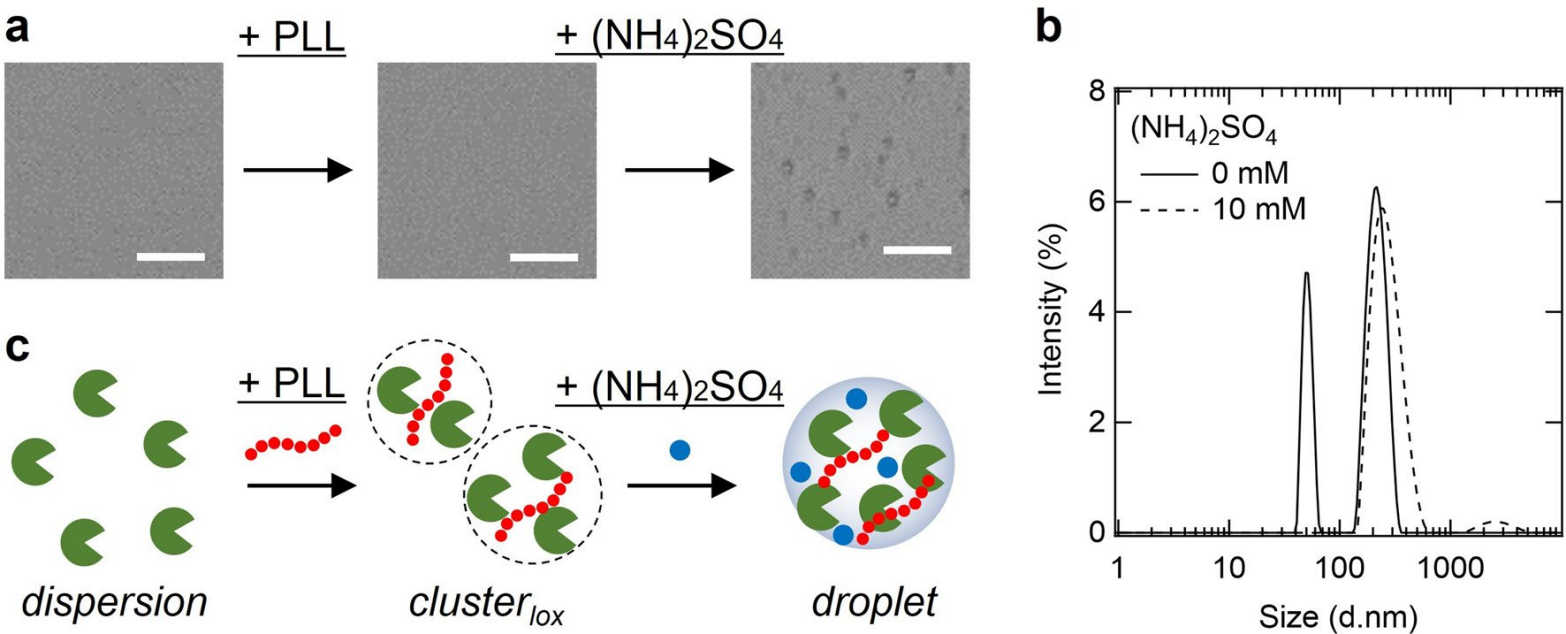
LOX assembly states in the presence of PLL and (NH_4_)_2_SO_4_. (**a**) Microscopy images of 0.1 µM LOX, 20 mM Tris–HCl (pH 8), 0 or 0.02 mM PLL (concentrations refer to lysine monomer units), 0 or 10 mM (NH_4_)_2_SO_4_. Scale bar, 10 µm (**b**) DLS data of sample solutions containing 0.1 µM LOX, 0.02 mM PLL, 20 mM Tris–HCl (pH 8), with 0 or 10 mM (NH_4_)_2_SO_4_. (**c**) Schematic image of assembly states of LOX with PLL and (NH_4_)_2_SO_4_.

We investigated the droplet formation conditions of LOX by adding scaffold molecules and salts (Fig. 1a). LOX has an isoelectric point at approximately pH 6 (calculated); hence, it is negatively charged at the physiological pH. PLL was used as a scaffold molecule for the formation of droplets with LOX because it has an isoelectric point at approximately pH 10 (calculated), and is a disordered structure at physiological pH^30^. It has been shown that salts play an important role in the formation of droplets via electrostatic interactions using synthetic polymers^29^. We investigated the effects of (NH_4_)_2_SO_4_ on the formation of LOX-PLL droplets because kosmotropic salts promote droplet formation^30^. Microscopic images 1 h after solution preparation showed no assembly in the presence of 0.1 µM LOX and 0.02 mM PLL, whereas droplets were observed in the presence of 0.1 µM LOX, 0.02 mM PLL, and 10 mM (NH_4_)_2_SO_4_ (Fig. 1a). We further investigated the effects of the salts on LOX-PLL droplets (Supplementary Fig. S1). In the presence of 25 mM sodium chloride (NaCl) and sodium thiocyanate (NaSCN), LOX-PLL did not form droplets. With increasing concentrations of NaCl and NaSCN, aggregate-like assemblies were observed above 100 mM. In contrast, in the presence of 25 mM sodium sulfate (Na_2_SO_4_) and (NH_4_)_2_SO_4_, LOX-PLL droplets were observed (Supplementary Fig. S1). These data indicate that a small amount of sulfate ions stabilizes the LOX-PLL droplets via the kosmotropic effect, which is consistent with a previous study^29^. We further investigated the mixing ratios of LOX and PLL (Supplementary Fig. S2 and S3) in the presence of (NH_4_)_2_SO_4_, indicating that 5 µM LOX and 1 mM PLL formed well-shaped and stable droplets in the presence of 5–20 mM (NH_4_)_2_SO_4_. Under these conditions, the droplets contained approximately 90% of the LOX in the solution (Supplementary Fig. S4).

Additionally, the presence of submicron-sized assemblies was investigated using dynamic light scattering (DLS) (Fig. 1b), because the size of these assemblies was below the detection limit of the optical microscope (~ 400 nm). In the absence of (NH_4_)_2_SO_4_, 0.1 µM LOX and 0.02 mM PLL formed two types of assemblies with diameters of approximately 50–60 and 200–300 nm, respectively, which could not be observed under the microscope (Fig. 1a,b). Here, we define these submicron-sized assemblies formed in the absence of (NH_4_)_2_SO_4_ as “clusters_lox_” (Fig. 1c). However, 0.1 µM LOX and 0.02 mM PLL with 10 mM (NH_4_)_2_SO_4_ formed broad size of droplets with diameters of approximately 200–500 nm (Fig. 1a,b), which could be observed under the microscope. The larger peak of cluster_lox_ overlapped with the peak of the droplet, suggesting that cluster_lox_ formed a part of the droplet-like assembly.

### Features of LOX-PLL droplets

**Figure 2:**
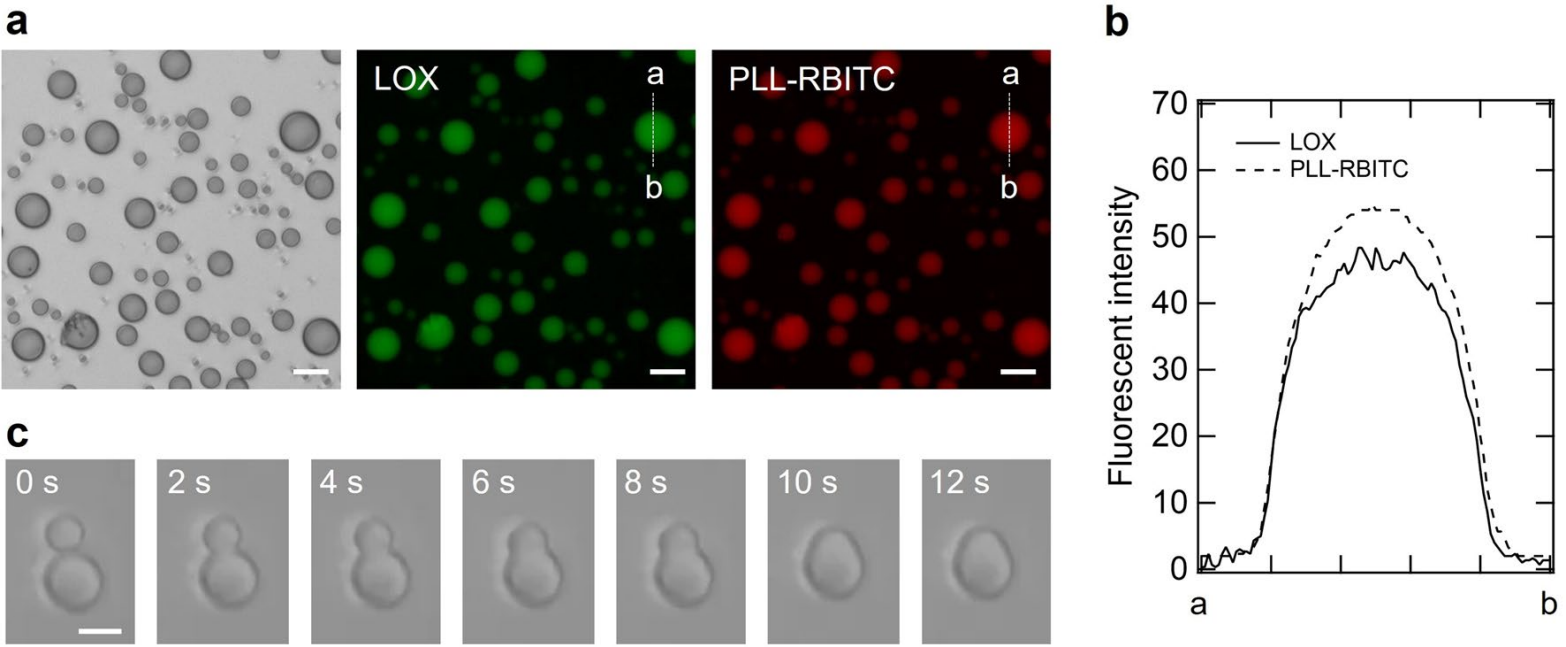
Features of LOX-PLL droplets. (**a**) Bright-field microscopic images of droplets (left) and fluorescent microscopic images of LOX (middle) and PLL-RBITC (right). The solution contained 5 μM LOX, 1 mM PLL, 6 mM (NH_4_)_2_SO_4_, 20 mM Tris–HCl, and 20 mM MES (pH 8). Scale bar, 20 μm. (**b**) Localization of LOX and PLL-RBITC in the droplet. Fluorescence intensity along the dashed white lines was quantified from the brightness of each pixel. (**c**) Bright-field microscopic images of coalescing LOX-PLL droplets. The solution contained 5 μM LOX, 1 mM PLL, 6 mM (NH_4_)_2_SO_4,_ 20 mM Tris–HCl, and 20 mM MES (pH 8). Scale bar, 10 μm.

We observed the LOX-PLL droplets using bright-field and fluorescence microscopy. Figure 2 shows microscopic images of the sample containing 5 μM LOX, 1 mM PLL, and 6 mM ammonium sulfate at pH 8. PLL was monitored using chemically modified Rhodamine B isothiocyanate (RBITC) (red), and LOX was monitored using an intrinsic flavin mononucleotide (green). Bright-field microscopic images showed droplets with spherical structures and diameters of 10 µm or less (Fig. 2a), indicating the typical appearance of liquid droplets. Fluorescence microscopy revealed that the droplets contained both LOX and PLL molecules uniformly, as indicated by green and red fluorescence (Fig. 2b). Furthermore, the LOX-PLL droplets coalesced in approximately 10 s (Fig. 2c), indicating that the LOX-PLL droplets have liquid-like fluidity. This coalescence also seems to occur in small droplets formed with 0.1 µM LOX because the particle size increased in solution with time (Supplementary Fig. S5). Moreover, because clusters_lox_ also tended to increase in particle size, it can be assumed that they have the same properties as droplets (Supplementary Fig. S5). To investigate the contribution of the electrostatic interaction between LOX and PLL in the formation of LOX-PLL droplets, we determined the effect of pH on the formation of LOX-PLL droplets (Supplementary Fig. S6). At pH 5, only amorphous aggregates were observed. At pH 6 and 7, both amorphous aggregates and liquid droplets were seen. At pH 8 and 9, however, only liquid droplets were observed. These results demonstrate that the electrostatic interaction between LOX and PLL plays a vital role in forming the liquid droplets because LOX and PLL have isoelectric points around pH 6 and 10, respectively.

### LOX activation in clusters_lox_ and droplets

**Figure 3:**
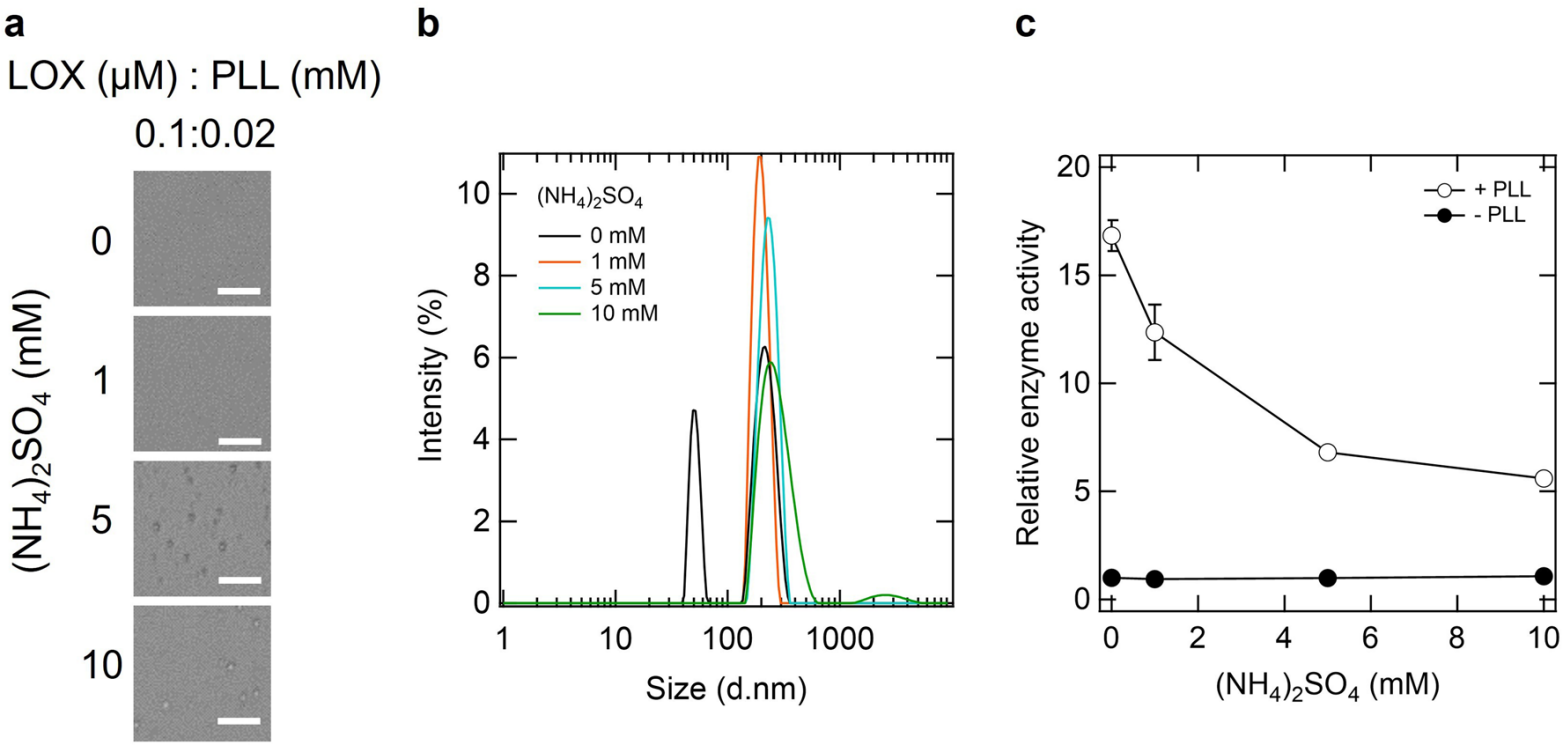
Assembly state and activity of LOX depend on the concentration of ammonium sulfate. (**a**) Bright-field microscopic images of enzyme assemblies. The solution contained 0.1 μM LOX, 0.02 mM PLL, 0–10 mM (NH_4_)_2_SO_4_, and 20 mM Tris–HCl (pH 8). Scale bar, 20 μm. (**b**) DLS data of sample solution containing 0.1 µM LOX, 0.02 mM PLL, and 20 mM Tris–HCl (pH 8), with 0–10 mM (NH_4_)_2_SO_4_. (**c**) Enzymatic activity of LOX in the presence of PLL and ammonium sulfate. Relative enzyme activity was defined as the initial reaction velocity in each condition divided by that in the absence of PLL and (NH_4_)_2_SO_4_.

We investigated changes in the assembly state and LOX activity depending on the concentration of ammonium sulfate. Microscopic images showed the formation of several micrometer-scale droplets above approximately 5 mM ammonium sulfate (Fig. 3a). Next, we investigated the presence of the LOX assembly using DLS measurements (Fig. 3b). Two peaks appeared in the absence of ammonium sulfate, as shown in Fig. 1c; the addition of 1 mM (NH_4_)_2_SO_4_ changed these two peaks into one large peak. With an increase in the concentration of (NH_4_)_2_SO_4_, this large peak shifted to the right and broadened (Fig. 3b). This result indicates that the size of the assembly increased with increasing ammonium sulfate concentration, which is consistent with the microscopic results (Fig. 3a). Under these conditions, we investigated how the enzyme activity of LOX changed depending on ammonium sulfate concentration (Fig. 3c). In the absence of PLL, the activity slightly increased depending on the ammonium sulfate concentration. In the presence of PLL and absence of ammonium sulfate, that is, in cluster_lox_, the enzyme activity of LOX was approximately 15 times higher (Fig. 3c). Although the activation rate decreased as the concentration of ammonium sulfate increased, it remained approximately five times more active in the presence of 10 mM ammonium sulfate (Fig. 3c). Furthermore, we confirmed that removing clusters_lox_ and droplets from solutions by centrifugation impaired LOX activity (Supplementary Fig. S7). The findings revealed that clusters_lox_ and droplets both increased LOX enzyme activity, with clusters_lox_ being more favorable for activation than droplets. The activating effects of LOX in the droplets and cluster_lox_ remained almost unchanged after 2 h and were retained at approximately 1 and sixfold for droplets and clusters_lox_, respectively, even after 24 h (Supplementary Fig. S8).

### Kinetic analysis of LOX in clusters_lox_ and droplets

**Figure 4:**
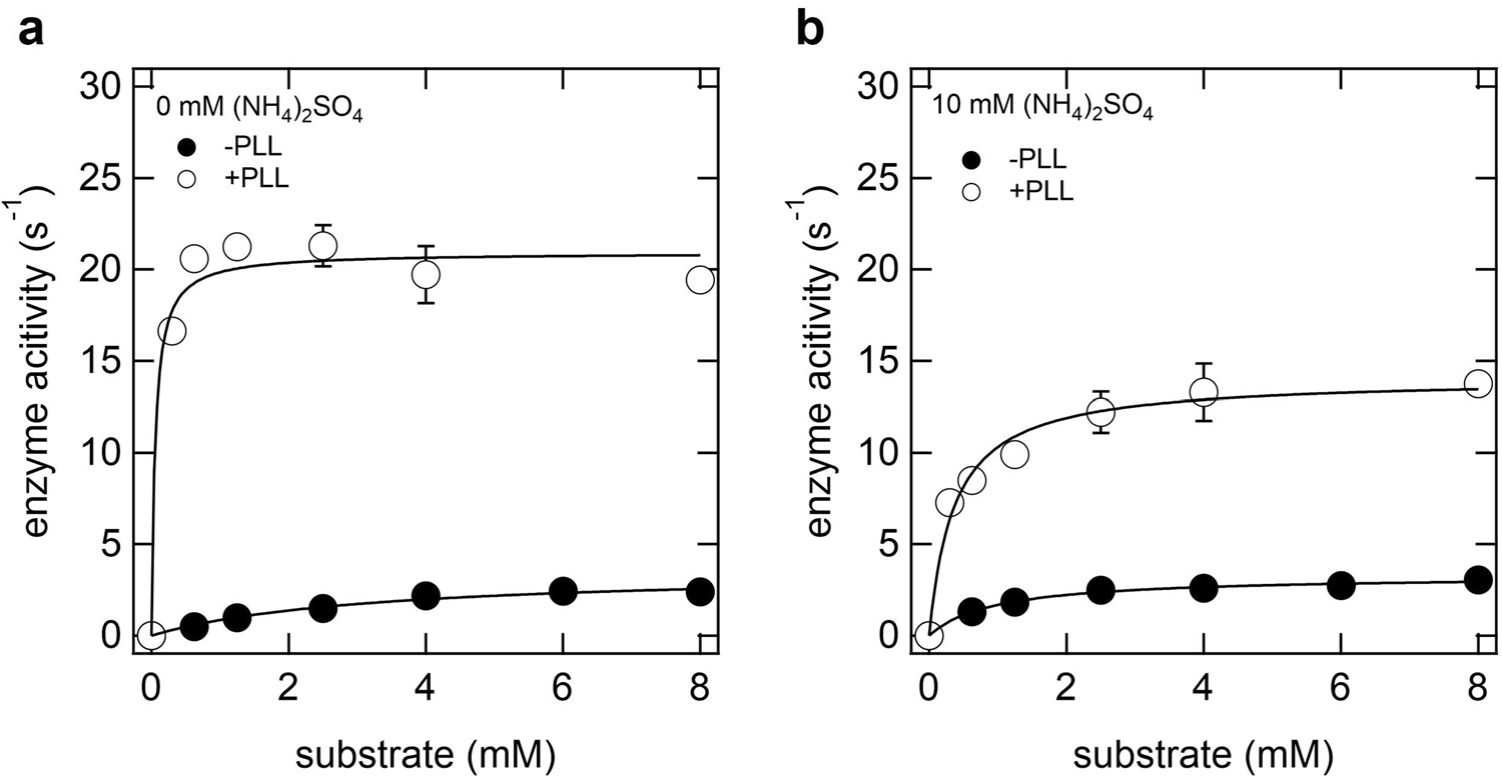
Enzyme kinetics of LOX in clusters_lox_. (**a**) and droplets (**b**). The sample solution contained 0.1 µM LOX, 0 or 0.02 mM PLL, 0–8 mM L-lactic acid, 20 mM Tris–HCl (pH 8), and 0 or 10 mM (NH_4_)_2_SO_4_.

To elucidate the detailed mechanism of enzyme activation by the formation of clusters_lox_ and droplets, we determined the kinetic parameters of LOX in 20 mM Tris–HCl (pH 8.0) at 25 °C (Fig. 4 and Table 1). The *K*_M_ of LOX in clusters_lox_ was 0.05 mM, approximately 64-fold smaller than that of LOX alone, indicating that cluster_lox_ is favorable for binding between LOX and its substrate (Fig. 4a). Additionally, the *k*_cat_ of LOX in clusters_lox_ was approximately sixfold higher than that in the dispersed state, indicating that the turnover number of LOX increased in clusters_lox_ (Fig. 4a). Next, the *K*_M_ of LOX-PLL in the droplet was 0.36 mM, approximately threefold smaller than that without PLL, indicating that the droplet is also favorable for binding between LOX and its substrate (Fig. 4b). The *k*_cat_ value of LOX in the droplets was approximately fourfold higher than that in the dispersed state (Fig. 4b). These results showed that the mechanism of enzyme activation in clusters_lox_ and droplets is common; the synergistic effect of an increase in substrate affinity and catalytic turnover; however, clusters_lox_ are more effective than droplets for LOX activation.

**Table 1:** Michaelis–Menten parameters for LOX in different assembly states.

Assembly state	0.02 mM PLL	10 mM (NH_4_)_2_SO_4_	*k*_cat_ (s^-1^)	*K*_M_ (mM)	*k*_cat_/*K*_M_
Disperse	–	–	3.58 ± 0.34	3.20	1.13
Disperse	–	+	3.29 ± 0.09	0.97	3.40
Clusters_lox_	+	–	20.92 ± 0.75	0.05	418.36
Droplets	+	+	14.06 ± 0.54	0.36	39.06

### Secondary structure of LOX in clusters_lox_

**Figure 5:**
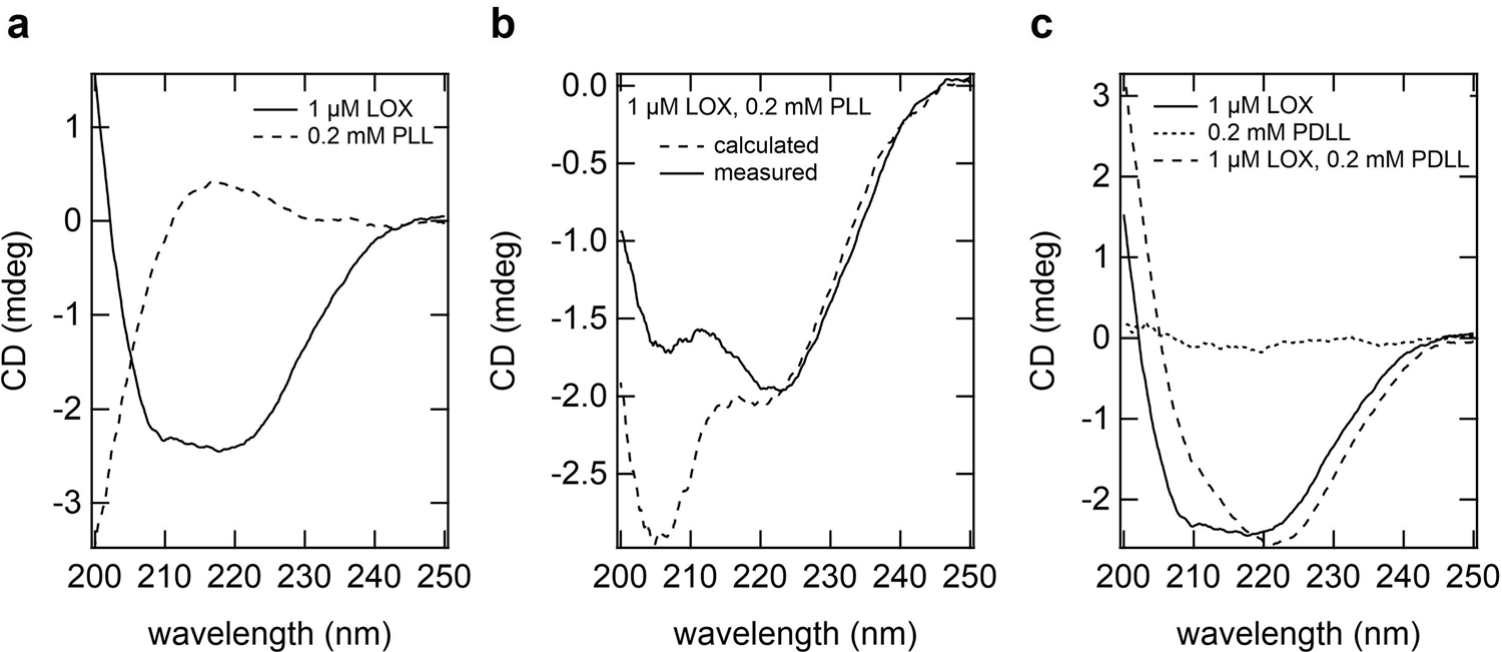
Far-UV CD spectra of LOX and PLL in clusters_lox_. (**a**) CD spectra of 1 μM LOX (solid line) and 0.2 mM PLL (broken line). (**b**) CD spectrum of LOX and PLL mixture (solid line), and CD spectrum calculated from that of LOX and PLL in A (broken line). (**c**) CD spectra of PDLL, LOX and LOX with PDLL.

The increase in *k*_cat_ may have resulted from the conformational change in LOX within clusters_lox_ and droplets. Thus, we investigated the secondary structure of LOX via far-ultraviolet (UV) circular dichroism (CD) spectroscopy. The far-UV CD spectra of LOX showed negative peaks at 208 and 218 nm, whereas the PLL spectra showed positive peaks between 210 and 230 nm (Fig. 5a). For samples forming clusters_lox_, the CD spectra of the mixture of LOX and PLL did not match those calculated from the individual spectra of LOX and PLL alone (Fig. 5b). These findings indicated that some structural changes in LOX and PLL were induced by their interactions.

PLL and LOX alone showed large far-UV CD ellipticity (Fig. 5a). Thus, we employed poly-(D,L)-lysine (PDLL), which also induces LOX droplets and activation (Supplementary Fig. S9) but is achiral in CD measurements to detect subtle changes in the secondary structure of LOX (Fig. 5c). As expected, PDLL showed almost no far-UV CD signal owing to its lack of optical activity (Fig. 5c). The spectrum of LOX drastically changed in the presence of 0.2 mM PDLL. Notably, the intensity of LOX at 210 nm was higher than that in the absence of PDLL. These results showed that a conformational change in LOX was induced in clusters_lox_, which may have caused an increase in *k*_cat_. For samples containing droplets, the CD spectra could not be obtained stably within the measurement time, probably due to droplet coalescence and sedimentation (data not shown).

### Interaction between substrate and PLL

Polymers with opposite charges to the substrate reduce *K*_M_^31^. The substrate l-lactic acid is negatively charged, hence, positively charged PLL may contribute to the decrease in *K*_M_. Therefore, we investigated the interaction of PLL and L-lactate by isothermal titration calorimetry (ITC) and nuclear magnetic resonance (NMR). The titration of L-lactate into the PLL solution showed no significant changes during titration (Supplementary Fig. S10a), indicating that L-lactate did not bind to PLL. To further confirm the binding between L-lactate and PLL, ^1^H-NMR spectrum of L-lactate was analyzed in the presence and absence of PLL (Supplementary Fig. S10b). L-lactate alone was assigned by three peaks ranging from 1.22–1.24, 1.34–1.38, and 1.48–1.50 ppm. The addition of PLL to the L-lactate solution did not change the intensities or chemical shifts of the peaks. These findings revealed that L-lactate does not interact with PLL. Thus, cluster_lox_ and droplet play an essential role in increasing the affinity between the substrate and enzyme, rather than between the substrate and PLL.

## Discussion and conclusions

In this study, we reported that the enzyme activity of LOX is increased by the formation of clusters_lox_ and droplets induced by the addition of PLL. The mechanism of LOX activation may be derived from kinetic parameters (Fig. 4 and Table 1) and conformational properties (Fig. 5). The enzyme activity of LOX increased in both clusters_lox_ and droplets, resulting from decreased *K*_M_ and increased *k*_cat_. The *k*_cat_ value of clusters_lox_ was higher than that of the droplets. In addition, the degree of activation decreased as the droplet size increased (see Fig. 3). This suggests that the degree of activation may not be uniform throughout the droplet, such that the LOX activity is higher near the droplet surface. In contrast, the *K*_M_ of the droplets decreased compared to the dispersed state, and it is likely that the compartmentalization effect of the substrate inside the droplets^10^. In addition to the compartmentalization effect, hundreds of nanometer-sized clusters_lox_ are more dispersible in solution than several micrometer-sized droplets; therefore, they probably have a higher probability of collision with the substrate. This may be the reason the *K*_M_ value of the clusters_lox_ was smaller than that of the droplets.

The structural change of LOX in the clusters_lox_ detected in CD is likely to be related to LOX activation, as LOX has been well investigated for its activity-structure relationship^32–35^. The LOX structure contains a core TIM barrel of eight α-helices and eight β-strands, binds FMN at the C-terminus of the β-barrel, and, importantly, has a very flexible loop and a short helix covering the active site^32^. Changes in this flexible loop structure and dynamics alter LOX activity by controlling substrate uptake and product release as shown in previous studies using mutations to this flexible loop^33–35^. The active site is open when this flexible loop adopts a disordered structure, and the active site is closed when it adopts a folded structure^33–35^. Specifically, in the crystal structure, α-helix decreases when active site is open compared to when active site is closed^33–35^. Thus, the decrease in α-helical content upon clusters_lox_ formation may reflect structural changes and/or dynamics in the flexible loop covering the active site (Fig. 5). As no method has been established to investigate the conformational changes of folded enzymes in the enzyme assemblies, investigation of detailed conformational changes of LOX will be a future challenge.

Changes in enzyme structure and/or dynamics that accompany cluster_lox_ and droplet formation can be attributed to the following possibilities. First, enzyme interaction with the polymer in cluster_lox_ and droplets may change the structural stability of the native enzyme, leading to an increased *k*_cat_^36,37^. Second, the enzyme assemblies like droplets are highly crowded with macromolecules, resulting in the exclusion of water molecules^38,39^. This crowding environment may stabilize non-native structures that differ from those in dilute conditions^40^. Therefore, crowding may promote the transition state of the enzyme, leading to enhanced enzyme activity. Finally, droplets represent a nonpolar environment compared to a dilute solution^39,41^. A nonpolar solution increases the stability of hydrophobic interaction^38,39,42^, hydrogen bonds, and electrostatic interactions^43^, which change enzyme activity. Further structural analysis of the enzyme in clusters_lox_ and droplets will be an interesting subject for future research.

The activation of LOX within the enzyme assemblies can provide valuable information for industrial applications. Currently, the primary methods of enzyme activation are protein engineering^44^ and directed evolution^45^. These methods generate favorable mutants with high activity through repeated mutagenesis and screening. However, it is time-consuming and costly to produce desirable mutants. In contrast, it is very simple to form an enzyme assembly using a polyelectrolyte to improve activity. LOX is an oxidoreductase applied to biofuel cells^46,47^ and biosensors^48^. Thus, the formation of enzyme assemblies represents a versatile approach for improving enzyme activity in practical applications using LOX or other enzymes.

## Methods

### Materials

Poly-L-lysine hydrobromide (MW, 70,000–150,000 Da), poly-(D,L)-lysine hydrobromide (MW, 25,000–40,000 Da), and 2,6-dichloroindophenol sodium salt hydrate (DCIP) were obtained from Sigma-Aldrich Co. (St Louis, MO, USA). NaCl, (NH_4_)_2_SO_4_, Na_2_SO_4_, NaSCN, and dimethyl sulfoxide (DMSO) were obtained from Kanto Chemical Co., Inc. (Tokyo, Japan). Tris(hydroxymethyl)aminomethane was obtained from Nacalai Tesque (Kyoto, Japan). 2-Morpholinoethanesulfonic acid monohydrate and 3-[4-(2-Hydroxyethyl)-1-piperazinyl] propanesulfonic acid (EPPS) were obtained from Dojindo Laboratories (Kumamoto, Japan). l-lactic acid was obtained from Tokyo Chemical Industry Co., Ltd. (Tokyo, Japan). Rhodamine B isothiocyanate (RBITC) was obtained from Santa Cruz Biotechnology (Dallas, TX). LOX was prepared as previously described^46^.

### Fluorescent labeling of PLL

PLL labeled with the amine-reactive dye RBITC (excitation/emission:555/580 nm) was prepared following the manufacturer’s instructions. Briefly, a solution of RBITC (1.77 mM) in DMSO (50 μL) was quickly added to a stirred solution of 20 mM PLL and 20 mM EPPS (950 μL; pH 8.5) at 25 °C. After the reaction mixture was gently stirred for 1.5 h, 200 mM Tris–HCl (100 μL, pH 8.5) was added. The PLL-dye conjugates were purified by filtration through Amicon Ultra-0.5 mL centrifugal filters with a molecular weight cutoff (MWCO) of 3 kDa (Millipore Sigma). The final concentration of PLL was determined using bicinchoninic acid (BCA) assay. The number of RBITC molecules conjugated to PLL in 10 mM Tris–HCl (pH 8.0) was determined from the absorbance at 556 nm, using the molar absorption coefficient ε_556_ = 87,000 M^−1^ cm^−1^. The number of dye molecules per PLL molecule was 0.2.

### Enzyme assays

Enzyme solutions containing 0.1 μM LOX, 0–0.2 mM PLL, and 0–10 mM ammonium sulfate in 20 mM Tris–HCl (pH 8) were prepared and left standing for 20 min. A 90 μL aliquot of enzyme solution was mixed with a 10 μL aliquot of substrate solution containing 0–80 mM L-lactic acid and 1 mM DCIP solution. The initial reaction velocities (*v*_0_) were determined from the slope of the initial decrease in absorbance at 555 nm using an Ultrospec 2100 pro UV/visible spectrophotometer (Amersham Biosciences Corp, Amersham, UK). Relative enzyme activity was defined as the initial reaction velocity under each condition divided by that in the absence of PLL and (NH_4_)_2_SO_4_. The *K*_M_ and *k*_cat_ values were determined by the initial reaction velocity on a theoretical Michaelis–Menten curve by nonlinear regression.

### Dynamic light scattering

Dynamic light scattering (DLS) experiments were performed using a Zetasizer Nano ZS light scattering photometer (Malvern Instruments, Worcestershire, UK) equipped with a 4 mW He–Ne ion laser (λ = 633 nm). To determine the size of LOX assemblies, solutions containing 20 nM LOX, 0–1 mM PLL, 0–10 mM (NH_4_)_2_SO_4,_ and 20 mM Tris–HCl were placed in a 1-cm path length disposable cuvette, and DLS measurements were performed at 25 °C at a detection angle of 173°. The viscosities of the solutions were approximated using water (η = 0.87 cP). All measurements were performed in 15 min after the solution preparation to prevent a decrease in scattering intensity by the sedimentation of enzyme assemblies. All the results are presented as the mean values of three independent experiments.

### Optical microscopy

Images were recorded using an all-in-one fluorescence microscope BZ-X710 (KEYENCE, Osaka, Japan) 1 h after sample preparations. Aliquots (100 μL) of the samples were placed in an ultra-low-attachment 96-well plate (Corning, NY, USA). All images were prepared using BZ-X Analyzer (KEYENCE).

### Isothermal titration calorimetry

Isothermal Titration Calorimetry (ITC) was performed using a Microcal Auto-iTC200 calorimeter (Malvern Instruments). The experiments consisted of a series of 0.2 μL injections of 4 mM PLL into 200 μL of 200 μM LOX solution or 1 mM l-lactic acid in the thermostatic cell with an initial delay of 60 s, a 0.4 s duration of injection, and a spacing of 120 s between injections. In all cases, the samples were dialyzed in the same buffer containing 20 mM Tris–HCl and 20 mM MES (pH 8) to minimize interference from mixing and dilution heat signals.

### Hydrogen-1 nuclear magnetic resonance spectroscopic analysis

Hydrogen-1 (^1^H) nuclear magnetic resonance (NMR) spectra were recorded in 20 mM Tris–HCl buffer at pH 8. The experiments were performed using a Bruker BioSpin Avance III 700 MHz NMR spectrometer at 25 °C.

### Circular dichroism

Circular dichroism (CD) experiments were performed in a 1-cm path-length quartz cuvette using a spectropolarimeter (J-720 W; JASCO Co., Ltd). For clusters_lox_ measurements, the enzyme solution containing 1 μM LOX and 20 mM Tris–HCl buffer (pH 8.0) was incubated with 0.2 mM PLL or 0.2 mM Poly-(D,L)-lysine at 25 °C for 20 min before measurement. The CD spectra of the samples were corrected by subtracting the corresponding spectra of buffers.

### Precipitation rate of LOX

The formation of liquid droplets of LOX with PLL was investigated based on the precipitation rate of the LOX. The concentration dependence of the PLL on liquid droplet formation was measured as follows: The PLL stock solution was prepared using 0–2 mM PLL in 20 mM Tris–HCl (pH 8). Aliquots (100 μL) of various solutions containing 1 mM LOX in 20 mM Tris–HCl (pH 8) were mixed with 100 μL of the PLL stock solution. Furthermore, the samples were centrifuged at 18,000×*g* for 20 min at 25 °C. The concentration of LOX in the supernatant was determined from the absorbance at 280 nm using an ND-1000 spectrophotometer (NanoDrop Technologies, Wilmington, DE, USA). Precipitation rates were calculated as follows:

$${\text{Precipitation}}\,{\text{rate}}\left( \% \right) = \left[ {1 - {\text{C}}_{{\text{n}}} /{\text{C}}_{0} } \right] \times 100\left( \% \right)$$

where C_0_ (mM) is the concentration in the supernatant without PLL and C_n_ (mM) is the LOX concentration in the supernatant with PLL.

## Supplementary Information

Supplementary Information.

## Data Availability

The data supporting the findings of this study are available in the paper and Supplementary file. All other data are available from the corresponding authors upon request.
