# Supplementary material for: Activation of L-lactate oxidase by the formation of enzyme assemblies through liquid–liquid phase separation
Source: Sci Rep. 2023 Jan 25;13:1435. doi: 10.1038/s41598-023-28040-1 (PMC9877012; doi:10.1038/s41598-023-28040-1)
Supplement: Supplementary file 1 — Supplementary Information. [file 41598_2023_28040_MOESM1_ESM.pdf]

Supplementary Information

Activation of L-lactate oxidase by the formation of enzyme assemblies through liquid–liquid phase separation

Tomoto Ura[1,2], Ako Kagawa[2], Nanako Sakakibara[1,2], Hiromasa Yagi[2], Naoya Tochio[2], Takanori Kigawa[2], Kentaro Shiraki[1,*], Tsutomu Mikawa[2,*]

[1]Faculty of Pure and Applied Sciences, University of Tsukuba, 1-1-1 Tennodai, Tsukuba, Ibaraki 305-8573, Japan.

[2]RIKEN Center for Biosystems Dynamics Research, 1-7-22 Suehiro-cho, Tsurumi-ku, Yokohama 230-0045, Japan

* To whom correspondence should be addressed. Tsutomu Mikawa. Tel: +81-45-633-8013; Fax: +81-45-503-9643; Email: mikawa@riken.jp

Correspondence should also be addressed to Kentaro Shiraki. Tel: +81-29-8535306; Fax: +81-29-8535215; E-mail: shiraki@bk.tsukuba.ac.jp

Supplementary Figure S1

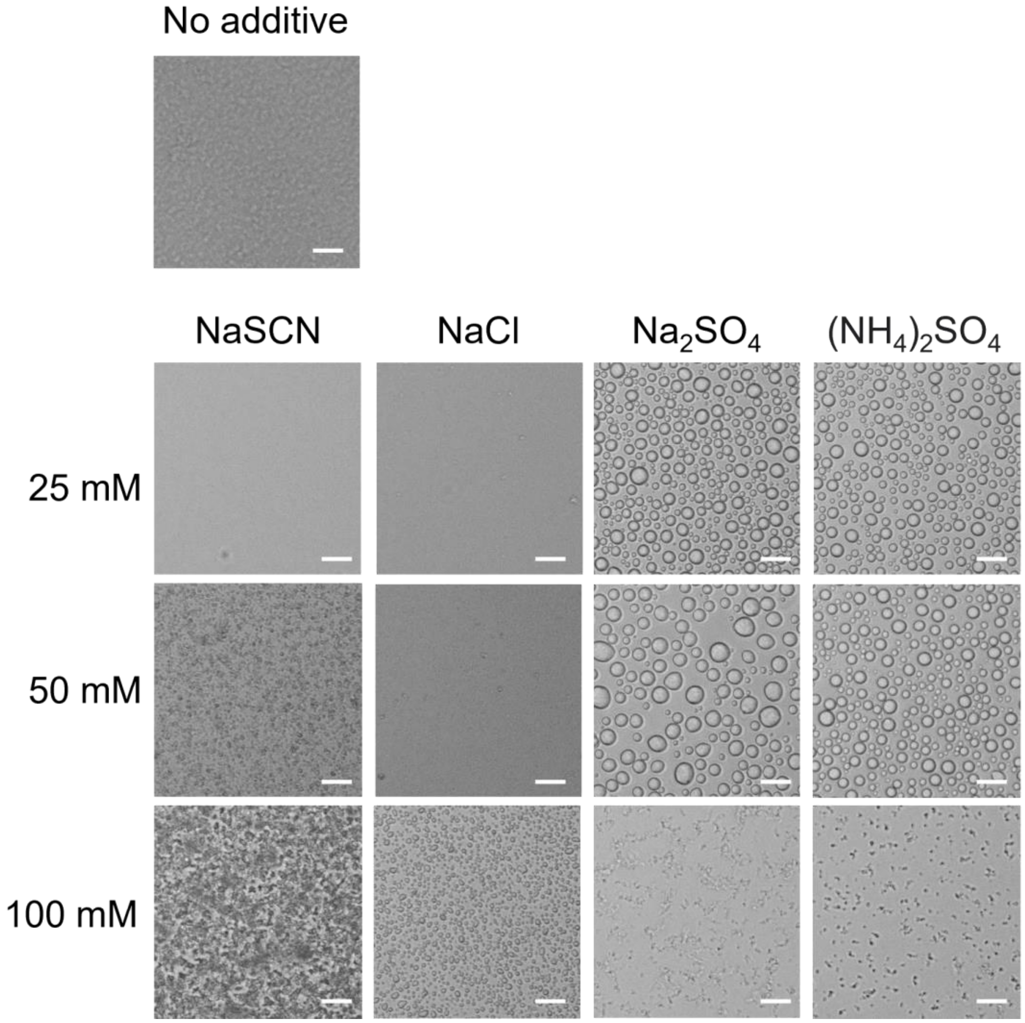

Fig. S1 Effect of the salt type and concentration on the formation of LOX-PLL assemblies. All solutions were prepared with 5 μM LOX, 1 mM PLL (concentrations refer to lysine monomer units), and salts (sodium thiocyanate: NaSCN, sodium chloride: NaCl, sodium sulfate: Na_2SO_4, ammonium sulfate: $((NH_4)_2SO_4)$ concentrations between 25–100 mM, and 20 mM Tris HCl (pH 8). Scale bar, 20 μm.

Supplementary Figure S2

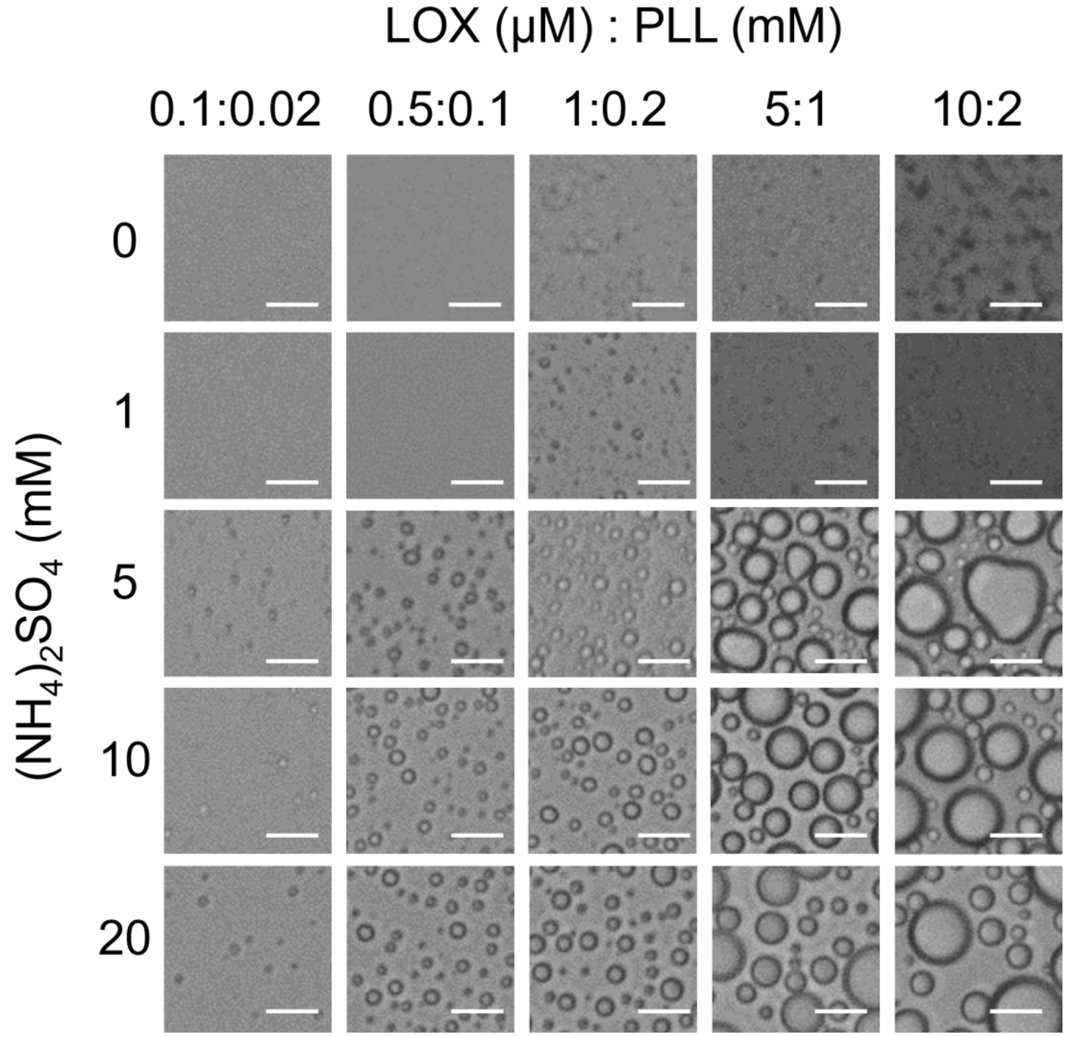

Fig. S2 Effect of ammonium sulfate on the formation of LOX-PLL assemblies. All solutions were prepared using 0.1–10 μM LOX, 0.02-2 mM PLL, 0–20 mM ammonium sulfate, and 20 mM Tris HCl (pH 8). Scale bar, 10 μm.

Supplementary Figure S3

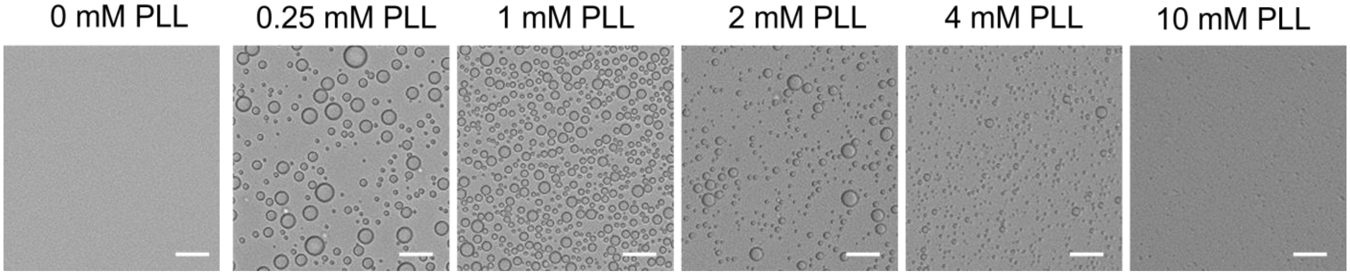

Fig. S3 Effect of PLL concentration on LOX-PLL droplet formation. All solutions were prepared with 5 μM LOX, 0–10 mM PLL, 10 mM $(NH_4)_2SO_4$, and 20 mM Tris-HCl (pH 8). Scale bar, 20 μm.

Supplementary Figure S4

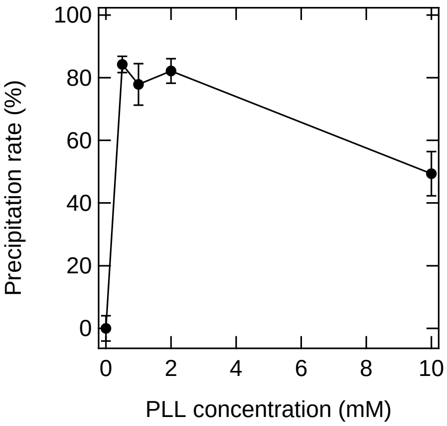

Fig. S4 Precipitation rate of LOX in the presence of PLL. All solutions were prepared with 5 μM LOX and PLL concentrations between 0 and 10 mM, 10 mM $(NH_4)_2SO_4$, and 20 mM Tris-HCl (pH 8).

Supplementary Figure S5

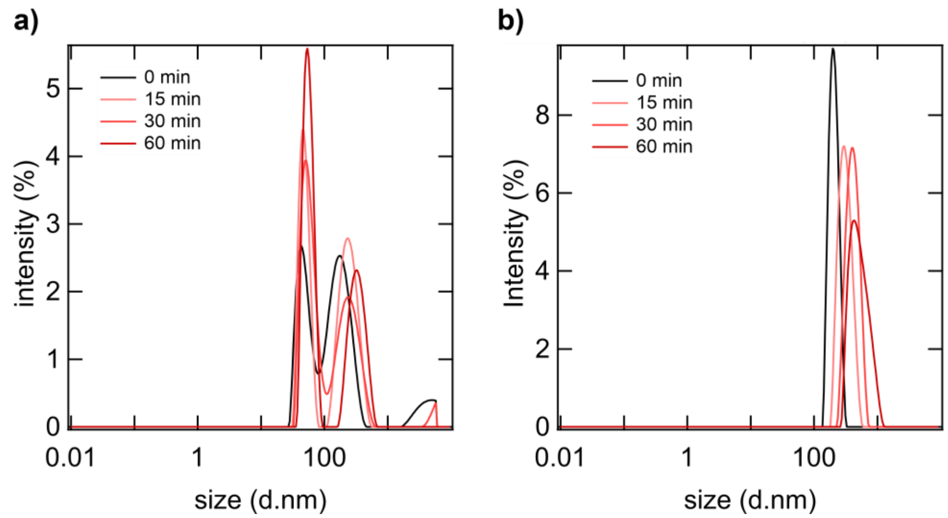

Fig. S5 Time-dependent changes in particle size of the LOX-PLL cluster **(a)** and droplet **(b).** All solutions contained 0.1 μM LOX, 0.02 mM PLL, 0 or 10 mM $(NH_4)_2SO_4$, and 20 mM Tris HCl (pH 8.0).

Supplementary Figure S6

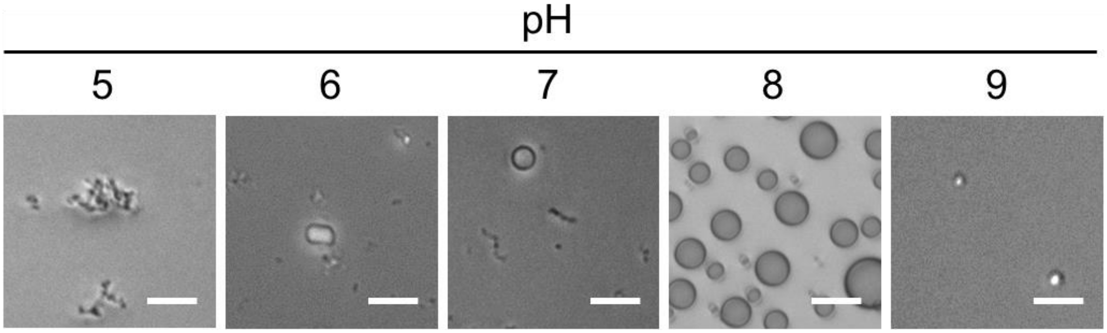

Fig. S6 Effect of PLL concentration on LOX-PLL droplet formation. All solutions were prepared with 5 μM LOX, 0–10 mM PLL, 10 mM $(NH_4)_2SO_4$, 20 mM MES, and 20 mM Tris-HCl (pH 5-9). Scale bar, 20 μm.

Supplementary Figure S7

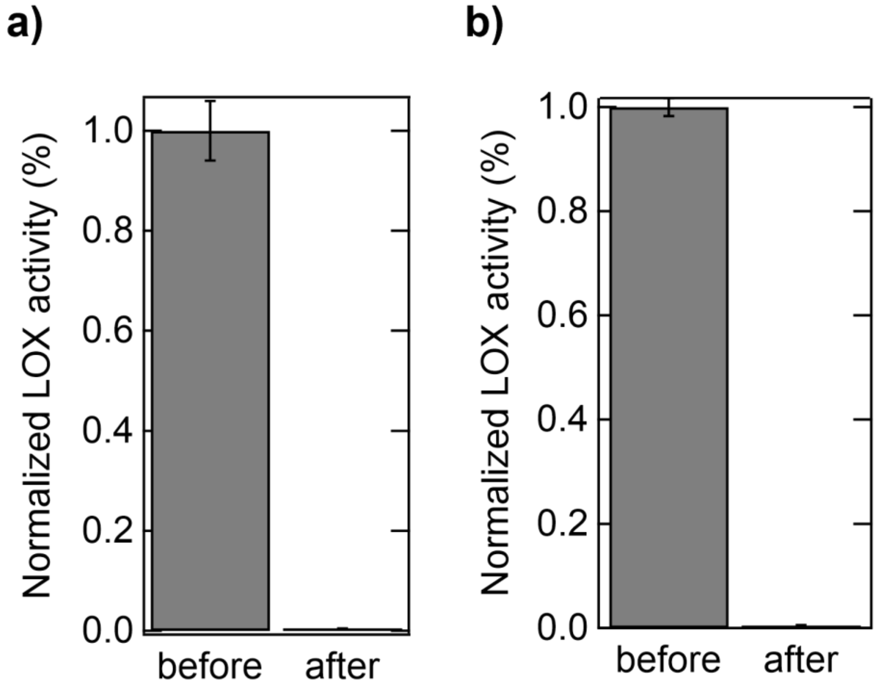

Fig. S7 Enzyme activity of LOX before and after centrifugation. **a**) Enzyme activities before and after the centrifugation of samples containing 0.1, 0.02 mM PLL, and 20 mM Tris HCl (pH 8) at 18,800 × g for 20 min. **b**) Enzyme activities before and after centrifugation of samples containing 0.1 μM LOX, 0.02 mM PLL, 10 mM $(NH_4)_2SO_4$, and 20 mM Tris HCl (pH 8) at 18,800 *g* for 20 min.

Supplementary Figure S8

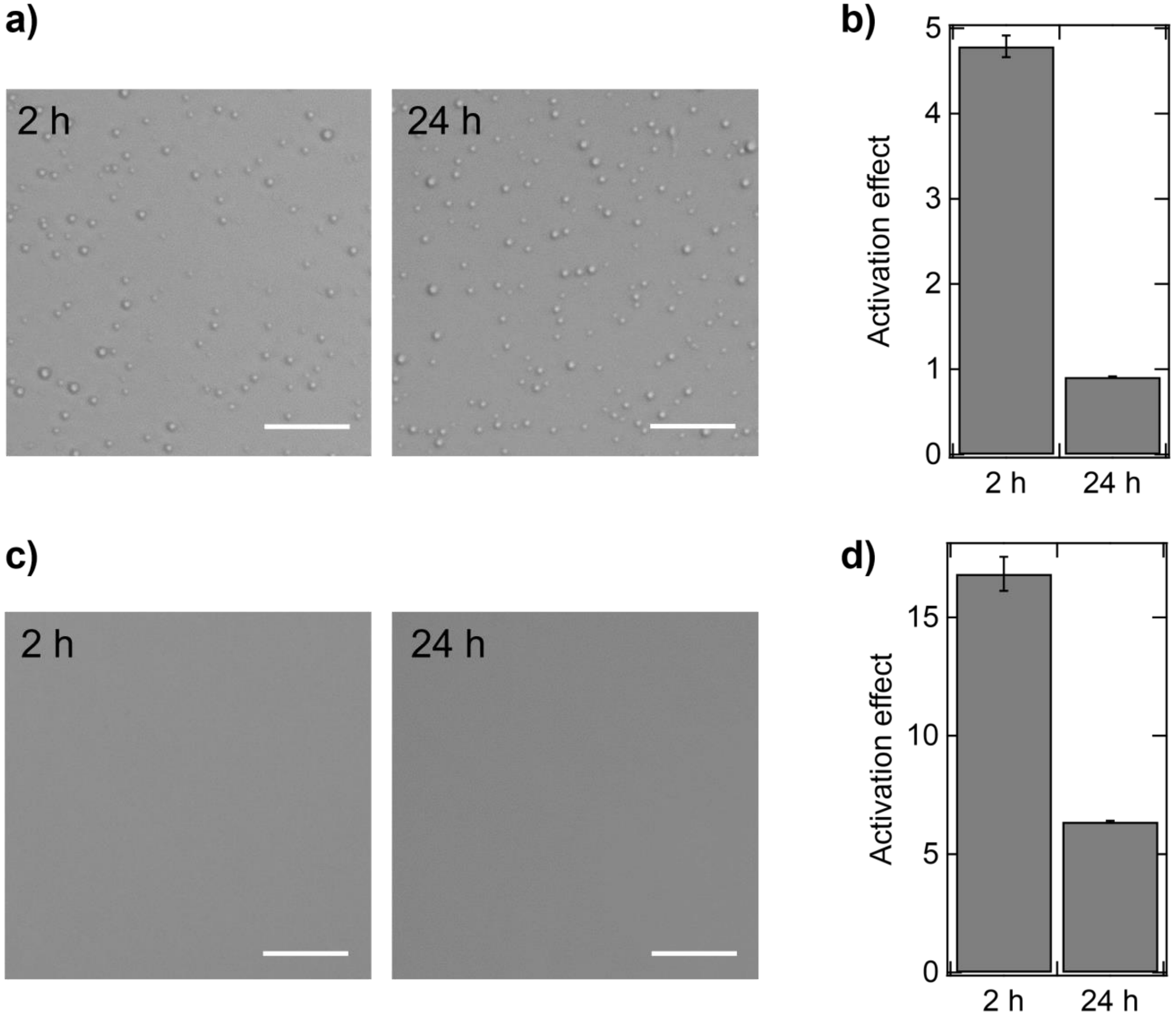

Fig. S8. Stability of droplets and clusters$_{lox}$. **a**) Bright-field field microscopic images of LOX-PLL droplets after 2 h and 24 h. The solution contained 0.1 μM LOX, 0.02 mM PLL, 10 mM $(NH_4)_2SO_4$, and 20 mM Tris HCl (pH 8). **b**) Activation effect of LOX in the droplets after 2 h and 24 h. The solution contained 0.1 μM LOX, 0.02 mM PLL, 10 mM $(NH_4)_2SO_4$, 0.1 mM DCIP, 1 mM L-lactic acid, and 20 mM Tris HCl (pH 8). **c**) Bright field microscopic images of cluster$_{lox}$ after 2 h and 24 h. The solution contained 0.1 μM LOX, 0.02 mM PLL, and 20 mM Tris HCl (pH 8). **d**) Activation effect of LOX in the cluster$_{lox}$ after 2 h and 24 h. The solution contained 0.1 μM LOX, 0.02 mM PLL, 0.1 mM DCIP, 1 mM L-lactic acid, and 20 mM Tris HCl (pH 8). Activation effect was defined as the initial reaction velocity in each condition divided by that in the absence of PLL and $(NH_4)_2SO_4$.

Supplementary Figure S9

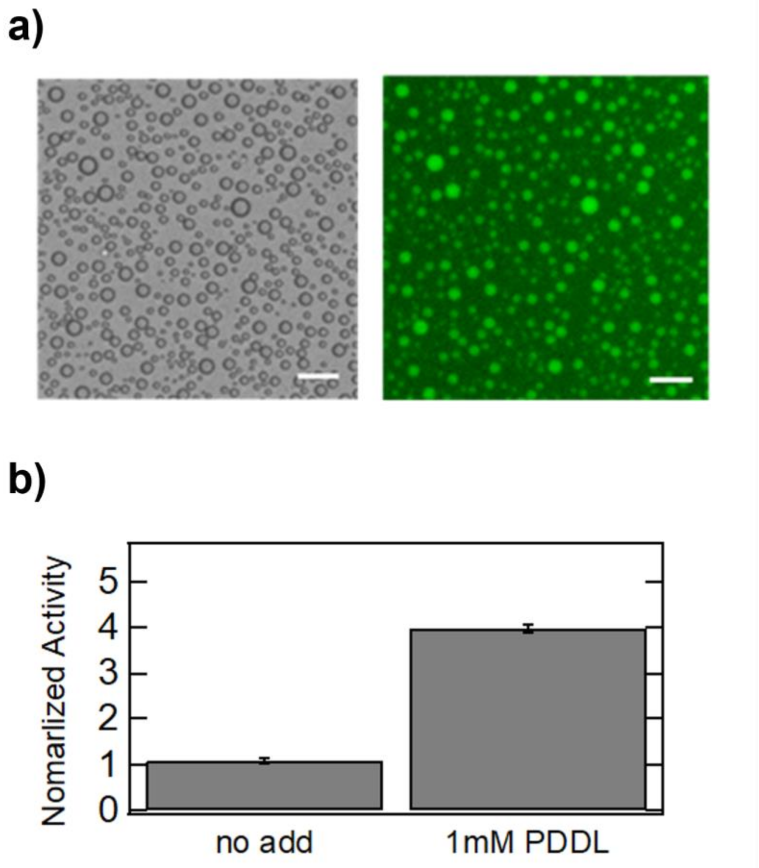

Fig. S9 Formation and activation of LOX-PDDL droplets. **a)** Blight field microscopic images of droplets (left) and fluorescent microscopic images of LOX (right). The solution contained 5 μM LOX, 1 mM PDLL, 6 mM $(NH_4)_2SO_4$, 20 mM Tris HCl, and 20 mM MES (pH 8). Scale bar, 20 μm. **b)** Normalized activity of LOX in the presence of 1 mM PDLL.

Supplementary Figure S10

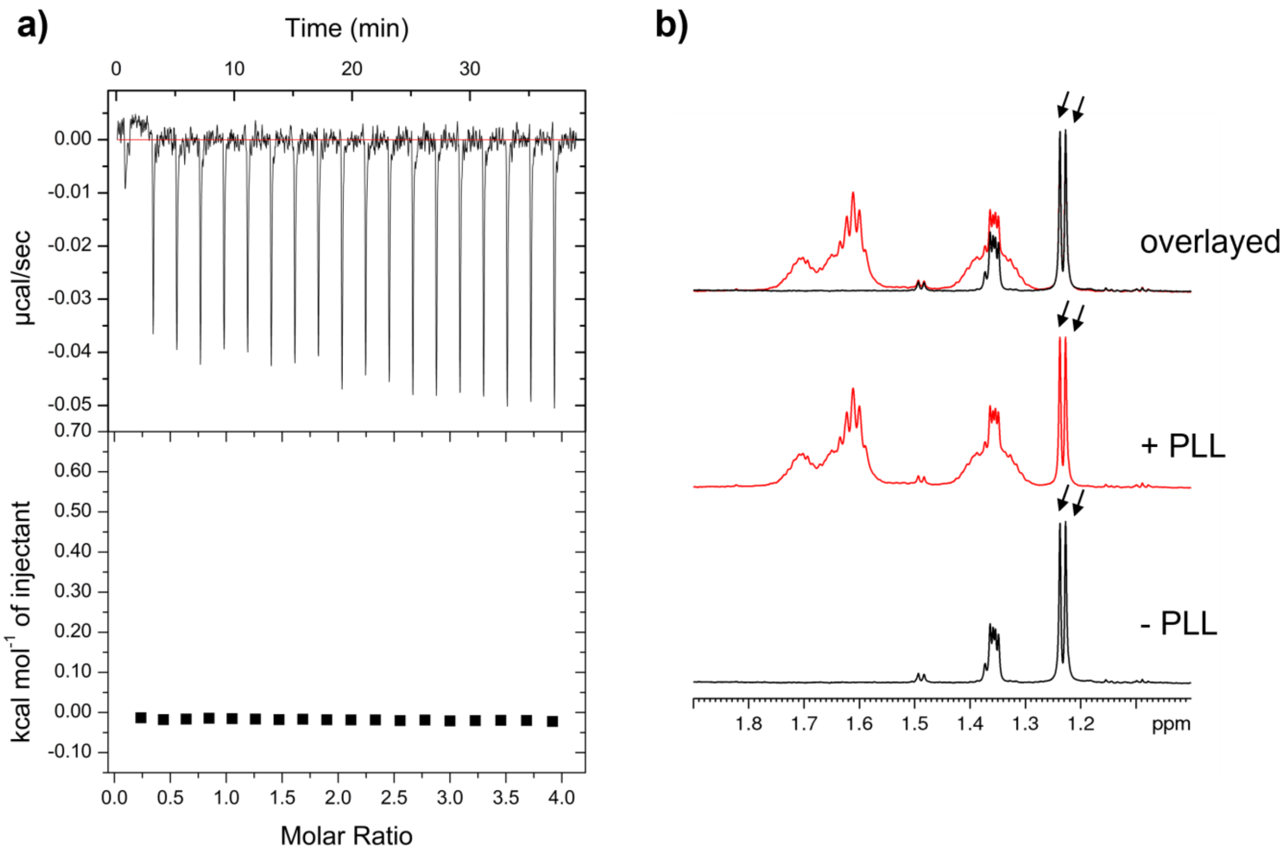

Fig. S10. Interaction of PLL with L-lactate. **a)** ITC analysis to quantify the interaction between PLL and L-lactate. **b)** 1H-NMR spectra of PLL and L-lactate in the presence and absence of the overlaid PLL.
